# The Role of Intestinal Microbiota in Regulating the Metabolism of Bile Acids Is Conserved Across Vertebrates

**DOI:** 10.3389/fmicb.2022.824611

**Published:** 2022-02-15

**Authors:** Fan Xiong, Sijia Chen, Ivan Jakovlić, Wenxiang Li, Ming Li, Hong Zou, Guitang Wang, Shangong Wu

**Affiliations:** ^1^Key Laboratory of Aquaculture Disease Control, Ministry of Agriculture, and State Key Laboratory of Freshwater Ecology and Biotechnology, Institute of Hydrobiology, Chinese Academy of Sciences, Wuhan, China; ^2^University of Chinese Academy of Sciences, Beijing, China; ^3^Bio-Transduction Lab, Wuhan Institute of Biotechnology, Wuhan, China

**Keywords:** gut microbiota, bile acid, regulation, fish, mammals

## Abstract

In mammals, bile acid (BA) concentrations are regulated largely by the gut microbiota, and a study has shown that some metabolic responses to the gut microbiota are conserved between zebrafish and mice. However, it remains unknown whether the influence of specific intestinal microbes on BA metabolism is conserved between higher and lower vertebrates (i.e., mammals and fish). In the present study, *Citrobacter freundii* GC01 isolated from the grass carp (*Ctenopharyngodon idella*) intestine was supplemented to the fish and mice feed. We found the changes in the bile acid profile, especially significant changes in secondary BAs in both grass carp and mice fed on *C. freundii*. Also, lipid metabolism was significantly affected by *C. freundii*. Analysis of liver transcriptome sequencing data and validation by RT-qPCR revealed that the *CYP7A1* gene was significantly up-regulated in both grass carp and mice. In addition, the overexpression of *HNF4B* from grass carp resulted in a significant increase in the expression level of *CYP7A1*. Generally, our results suggest that the metabolism of BAs by intestinal microbiota is conserved across vertebrates. Furthermore, specific intestinal bacteria may regulate the bile salt synthesis through *CYP7A1* and that *HNF4B* might be an important regulator of BA metabolism in fish.

## Introduction

Obesity and obesity-related disorders, including hypertension, cardiovascular pathology, type II diabetes, and non-alcoholic fatty liver disease, have become a serious epidemic threat to humans (Bäckhed et al., 2004). This puts efforts to determine the host and environmental factors that affect the energy harvest from the diet into scientific focus. The gastrointestinal tract of vertebrates is a complex ecosystem that usually harbors a diverse bacterial community (Björkstén, 2006). During the evolution of both the gut microbiota and the host, the symbiotic microbial community has become an integral component of the host, exquisitely tuned to the host’s physiology, and capable of performing metabolic functions that hosts did not manage to evolve on their own (Bäckhed et al., 2004; Rawls et al., 2004; Ley et al., 2008). Among its many important functions, the gut microbiome can convert feed stuff into microbial biomass and fermentation end products that can be utilized by the animal host (Flint et al., 2008; Kong et al., 2010). Certain microbial taxa have been associated with obesity (for example, Bacteroidetes, and Firmicutes), giving rise to the term “obese gut microbiome” for its increased capacity to harvest energy from the diet (Turnbaugh et al., 2006). This suggests that gut microbiota is an environmental factor that regulates fat storage. Using *in vivo* imaging of fluorescent fatty acid analogs delivered to gnotobiotic zebrafish hosts, it was found that microbiota stimulated the fatty acid uptake and lipid droplet formation in the intestinal epithelium and liver, which provides mechanistic insight into how microbiota-diet interactions regulate the host energy balance (Semova et al., 2012). However, lipid metabolism is very complex, and gut microbiota synthesize thousands of molecules, many of which influence host physiology, so the impact of microbiota on intestinal metabolism of dietary fat remains only partially understood.

Bile acids (BAs) are essential for dietary lipid absorption (Watanabe et al., 2006), so disruption of bile acid homeostasis can affect lipid homeostasis by changing lipid absorption and altering the expression of genes that control the lipid metabolism (Kim et al., 2007). In the process named enterohepatic circulation, primary bile acids are synthesized in the liver, metabolized by intestinal microbiota into secondary bile acids, which are eventually reabsorbed and returned to the liver (Romano et al., 2020). Recent studies have shown that the gut microbiome has important roles not only in adjusting the levels and profiles of the bile acid pool but also in influencing the expression of genes, which is mostly achieved through the bile acid-activated farnesoid X receptor (FXR) (Kim et al., 2007). FXR is an evolutionarily conserved transcription factor that, upon activation by binding with bile salts, regulates a large number of genes involved in bile salt, lipid, and glucose metabolisms (Kim et al., 2007; Lefebvre et al., 2009; Wen et al., 2021). Secondary bile acids, such as deoxycholic acid (DCA) and lithocholic acid (LCA), are agonists of FXR (Vallim et al., 2013). They also negatively influence the biosynthesis of primary bile acids (Swann et al., 2011; Sayin et al., 2013). Although the mechanism by which the intestinal microbiota regulate bile acid concentrations remains to be fully elucidated, studies in model mammals (rats and mice) have suggested that the microbiome is an important modulator of bile acid metabolism and function (Swann et al., 2011; Sayin et al., 2013). The genome of zebrafish possesses orthologs of many mammalian genes known to be involved in bile salt homeostasis, including the *FXR* (named *NR1H4* in fish) (Wen et al., 2021), and a study has shown that some metabolic responses to the gut microbiota are conserved between zebrafish and mice (Rawls et al., 2004). However, it remains unknown whether the influence of intestinal microbiota on bile acid metabolism is also conserved in lower vertebrates, such as fish.

*Citrobacter freundii* is a common species in the fish intestine, and it can be easily cultured in a high-fat culture medium (Zhang et al., 2020). Although *C. freundii* is generally regarded as an opportunistic pathogen for fish, commonly it is not pathogenic; quite the contrary, at low abundance it can enhance the host’s energy harvest (Xiong et al., 2020; Zhang et al., 2020). For example, Zhang et al. (2020) found that the addition of *C. freundii* to a high-fat diet increased the lipid accumulation in mesenteric adipose tissue in Nile tilapia (*Oreochromis niloticus*); they proposed that administration of *C. freundii* influenced the composition of intestinal microbiota and increased the efficiency of triglyceride absorption and transportation as the likely mechanism for increased lipid accumulation.

However, as the intestinal microbiome is an important modulator of bile acid metabolism, and bile acids are essential in dietary lipid absorption, we hypothesized that the actual mechanism behind this observation is associated with the impact of *C. freundii* on bile acid metabolism. To test this hypothesis, for this study we isolated *C. freundii* GC01 from the grass carp (*Ctenopharyngodon idella*) intestine and supplemented it to the fish and mice feed. The objectives were to (1) elucidate the influence of *C. freundii* GC01 on the bile acid metabolism, and (2) disclose whether the function of intestinal bacteria in the regulation of bile acid metabolism is conserved among lower (fish) and higher (mammals) vertebrates.

## Materials and Methods

### Animal and Feeding Trial

The culturing of grass carp, dietary supplementation of *Citrobacter freundii* GC01, and sampling strategy have been described before (Xiong et al., 2020). Briefly, juvenile grass carp (*n* = 180, weight = 40 ± 5 g) were divided into three groups and fed three different diets for 8 weeks: a standard diet as the control (Ctrl) group, a diet supplemented with 0.5*10^7^ cfu/g *C. freundii* GC01 (LD group), and a diet supplemented with 10^7^ cfu/g *C. freundii* GC01 (HD group). The doses of bacteria used here were selected on the basis of experiments on tilapia (Sheng, 2018). *C. freundii* GC01 was sprayed on the surface of the granulated feed and stored at 4°C until use to minimize the mortality rate of bacteria. At the end of the feeding trial, five fish specimens from each group were sampled randomly and dissected to collect hindgut contents (used to study intestinal microbiota). Five liver samples and hindgut contents from each group were sampled randomly and used for transcriptome sequencing and bile acid determination, respectively.

C57BL/6 mice (male, 3 weeks old) were purchased from the Hubei Center for Disease Control and Prevention and housed in specific pathogen-free environments under a controlled condition of 12 h light/12 h dark cycle at 20-22°C and 45 ± 5% humidity, with free access to chow and ultrapure water. Mice were randomly divided into three groups (each group containing three cages, and each cage containing five mice, as biological replicates). All three groups were fed a standard diet (10% fat calories) for 8 weeks, but supplemented with varying levels of *C. freundii* GC01: (1) Control group = no *C. freundii* supplementation (Ctrl group); (2) LD group = 10^6^ cfu/g *C. freundii*; (3) HD group = 10^8^ cfu/g *C. freundii*. The body weights and food intake were recorded once a week during the experiments. At the end of the feeding trial, the cecal content of three randomly selected mice from each cage was sampled for microbiota analysis. In addition, livers were carefully dissected and collected. All samples were flash-frozen in liquid nitrogen and long-term stored at −80°C.

### 16S rRNA Amplicon Sequencing Library Preparation

DNA was extracted using QIAamp Fast DNA Stool Mini Kit (Qiagen, Germany). NanoDrop 2000 Spectrophotometer (Thermo Scientific, Waltham, MA, United States) was used to check the concentration and quality of the extracted DNA. Extracted DNA was diluted to 10 ng/μL and stored at −80°C for downstream use. Universal primers 515F (5′-GTGYCAGCMGCCGCGGTA-3′) with a 12 nt. unique barcode at 5′-end and 806R (5′-GGACTACHVGGGTWTCTAAT-3′), were used to amplify the hypervariable V4 region of the bacterial 16S rRNA gene (Caporaso et al., 2011). Each sample was amplified in triplicate, in a 25 μL reaction mix containing 2xGoTaq Green Master Mix (Promega, Madison, WI, United States), 1 μM of each primer, and 10 ng genomic DNA. The PCR program included 5 min at 94°C, followed by 23 cycles of 94°C for 30 s, 50°C for 30 s, and 72°C for 30 s, and finally 10 min at 72°C. PCR products were purified using the MinElute 96 UF PCR Purification Kit (Qiagen, Valencia, CA, United States). All samples were sequenced using an Illumina Hiseq platform (HiSeq Reagent Kit V2, 500 cycles) by Novogene Biotechnology Co., Ltd. (Beijing).

### Analysis of the 16S rRNA Amplicon Sequencing Data

The raw sequence data were processed using QIIME Pipeline-Version 1.8.0^1^ (Caporaso et al., 2010). Overlapping paired-end reads were merged using the FLASH-1.2.8 software (Magoc and Salzberg, 2011). Only the merged sequences with high-quality reads (length > 250 bp, without ambiguous bases BN, and average base quality score > 30) were used for further analyses. All sequences were trimmed and assigned to each sample on the basis of their barcodes (barcode mismatches = 0). Chimeras were removed using the Uchime algorithm (Edgar et al., 2011). Non-chimera sequences were subsampled to the same sequence depth (11,784 reads per sample) using daisychopper.pl (Gilbert et al., 2009). This subset of sequences was clustered into OTUs (Operational Taxonomic Units) at a 97% identity threshold using CD-HIT (Li and Godzik, 2006). Singleton sequences were filtered out. OTU identities were assigned using the Greengenes database (release 13.8) (DeSantis et al., 2006) and UCLUST (Edgar, 2010). Sequences classified as unassigned and C_Chloroplast were removed. Alpha diversity (Chao1, PD_whole_tree, Shannon and Simpson index) and beta diversity (unweighted UniFrac metric and weighted UniFrac metric) indices of bacterial communities were calculated. Principal coordinate analysis (PCoA) was used to visualize similarities between groupings with unweighted_unifrac and weighted_unifrac distances (Navas-Molina et al., 2013). PERMANOVA analysis was performed to test for significant differences between groups in overall microbial composition with unweighted_unifrac and weighted_unifrac distances (Kelly et al., 2015). LEfSe (Linear discriminant analysis Effect Size) was determined using the Galaxy web tool^2^ to identify taxa associated with each experimental group (Segata et al., 2011; Afgan et al., 2018). PICRUSt analysis was conducted to predict metabolic functional profiles of gut microbiota after importing the normalized bacterial OTUs table in another Galaxy tool, PICRUSt, according to the KEGG database (Langille et al., 2013). Functional categories at level II, derived from the KEGG database modules (Kanehisa and Goto, 2000), were further analyzed using the STAMP software package (Parks et al., 2014).

### The Whole Genome Sequencing of *Citrobacter freundii* GC01

High quality DNA of *C. freundii* GC01 was used to construct a sequencing library. The whole-genome sequencing was performed using Illumina and Nanopore sequencing technologies. After removing the low-quality reads, the genome was assembled using Unicycler (0.4.8) software (Wick et al., 2017). First, the high-quality genomic contigs were assembled using high-accuracy Illumina data, and then the high-quality contigs were connected into a complete genome using Nanopore data. Thereafter, Pilon (1.22) software (Walker et al., 2014) was used to further improve the genome assembly using Illumina data. The protein-coding genes of the assembled genome were predicted by Prokka (1.1.2) software (Seemann, 2014). Seven databases, including UniProt (UniProt Consortium, 2019), KEGG (Kanehisa et al., 2008), GO (Harris et al., 2004), RefSeq (O’Leary et al., 2016), Pfam (El-Gebali et al., 2019), COG (Galperin et al., 2021) and Tigrfams (Haft et al., 2003), were used to obtain comprehensive gene function information.

### Analysis of the Liver Transcriptome Sequencing Data

The total RNA was extracted from livers of grass carp using RNeasy Mini Kit (Qiagen, Germany) according to the manufacturer’s protocol. RNA concentration was measured using the Qubit RNA assay kit (Life Technologies, Carlsbad, CA, United States), and integrity was assessed with the RNA Nano 6,000 assay kit (Agilent Technologies, Santa Clara, CA, United States). RNA of sufficiently high quality was used for library construction. Sequencing libraries were generated using the NEB Next Ultra RNA library prep kit for Illumina (New England Biolabs, Ipswich, MA, United States) following the manufacturer’s protocol. Libraries were sequenced on an Illumina Hiseq 4,000 platform and 150 bp pair-end reads were generated. Raw data reads in the fastq format were initially processed using in-house *perl* scripts. In this step, clean data (clean reads) were obtained by removing the adapter, poly-N, and poor quality data. The Q30 and GC contents of the clean data were calculated, and all downstream analyses were performed using the clean high-quality data. Clean data were mapped to the grass carp reference genome (Wang et al., 2015) using Hisat2 software (Kim et al., 2015). StringTie v1.2.3 software was used for the transcript assembly and to count the number of reads mapped to each transcript (Pertea et al., 2015). The transcripts were subjected to BLASTX similarity searches against the UniProt database with an *E*-value threshold of 10e5. UniProt accession IDs were used for the functional annotation of transcripts. Differential expression analysis of two groups/conditions was performed using the Ballgown package (Pertea et al., 2016), and the fragments per kilobase of exon per million fragments mapped (FPKM) were calculated for each transcript on the basis of the length of the transcript and the number of reads mapped to the transcript (Pertea et al., 2016). The resulting *p*-values were adjusted using the Benjamini and Hochberg approach for controlling the false discovery rate. Transcripts with an adjusted *p*-value < 0.05 (q value < 0.05) were designated as differentially expressed genes (DEGs). The KEGG annotation analysis was performed with the DAVID v6.8 platform (Huang et al., 2009). KEGG terms with corrected *p* < 0.05 were considered to indicate statistical significance.

### Validation of Differentially Expressed Genes by the Real-Time Quantitative PCR (qPCR)

To confirm the reliability of data obtained by RNA-Seq, DEGs associated with the lipid metabolism identified in the grass carp liver were selected for validation by qPCR, both in grass carp and mice liver samples. The primers used for qPCR are listed in Supplementary Table 1. qPCR was performed with iQ™ Universal SYBR^®^ Green Supermix (Bio-Rad Laboratories, Hercules, CA, United States) in a CFX96 Touch™ Real-Time PCR Detection System (Bio-Rad Laboratories, Hercules, CA, United States). Each qPCR mixture contained 0.8 μL of forward and reverse primers (for each primer), 1.0 μL template, 10.0 μL 2 × SYBR green master mix (TOYOBO, Japan), and 7.4 μL ddH2O. Each sample was run in triplicate and the β*-actin* gene was used as an internal control to normalize gene expression. The program for qPCR was as follows: 95°C for 10 s, 40 cycles of 95°C for 15 s, 55°C for 15 s, and 72°C for 30 s. Relative expression levels were calculated using the 2^–ΔΔ*Ct*^ method. Negative controls were set up by replacing the template DNA with dH2O to eliminate the possibility of DNA or primer dimer contamination. Data are presented as the mean ± standard deviation of three technical replicates. Differences were analyzed by the independent-sample *t*-test using SPSS 16.0 (IBM Corporation, Armonk, NY, United States). Throughout the study, *p*-values < 0.05 are referred to as statistically significant, unless if specified otherwise.

### Oil Red-O (ORO) Staining of the Frozen Liver Sections

To confirm the results from transcriptome analysis, liver sections were stained with ORO to detect lipids in the liver. Frozen sections of the livers were stained with ORO to detect lipid droplets and counterstained with Mayer’s hematoxylin (Luna, 1968). The percentage of ORO-positive areas to the internal surface was measured with a computer-assisted morphometry system (Image Pro Plus 6.0, Maryland, MD, United States). Data for each group are presented as the mean ± standard deviation. A *T*-test was performed to compare the abundance of lipid droplets between two groups.

### Biochemical Analysis

Serum triglycerides and total cholesterol levels were measured by the enzymatic colorimetric assay using a commercial assay kit (Nanjing Jiancheng Bioengineering Institute, China) according to the manufacturer’s protocol. All assays were performed in triplicate. A *T*-test was performed to compare the abundance of lipid droplets between pairs of groups.

### Biliary Acids Measurement and Statistics

Bile acids were extracted from intestinal content according to the method described by Hagio et al. (2011) and quantified by the high-performance liquid chromatography-mass spectrometry (HPLC-MS) as described elsewhere (Zhang et al., 2017). The total BAs were calculated as the sum of the concentrations of all measured BAs. Pairwise comparisons of the concentrations of measured BAs between pairs of groups were conducted using *T*-tests.

## Results

### Changes in the Gut Microbiota of Grass Carp and Mice

We set up three groups: control group (no *C. freundii* supplementation), LD: a low dose *C. freundii* supplementation group, and HD: a high dose *C. freundii* supplementation group. Detailed changes induced by the *C. freundii* GC01 dietary supplementation on the gut microbiota of grass carp have been described elsewhere (Xiong et al., 2020). Generally, grass carp fed with *C. freundii* showed a significant decrease in bacterial diversity compared to the controls (*p-*value < 0.05). The supplementation of *C. freundii* had a significant impact on the bacterial community structure (*p-*value = 0.001). A significant perturbation of the relative abundance of bacteria were observed in the orders Enterobacteriales, Pasteurellales, and Neisseriales; and genera *Adlercreutzia* (Actinobacteria), *Chryseobacterium* (Bacteroidetes), *Enterococcus* (Firmicutes), and *Citrobacter* (Proteobacteria). As expected, *Citrobacter* exhibited the highest relative abundance in the HD group (fed a high dose of *C. freundii*). PICRUSt-prediction revealed significantly more abundant genes associated with lipid metabolism in both experimental groups, and energy metabolism in the HD group in comparison to the control samples (*p-*value < 0.05) (Supplementary Figure 1).

**FIGURE 1:**
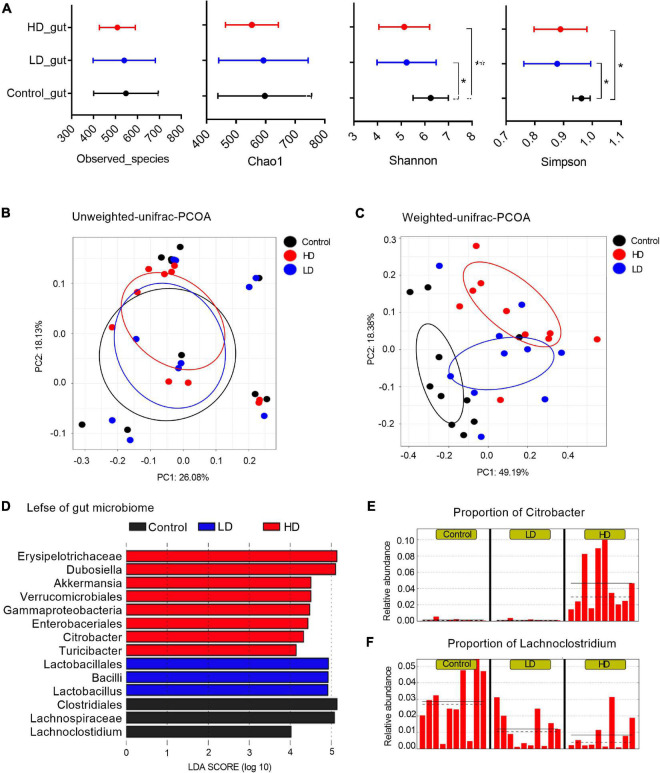
The mice gut microbiome in three groups. **(A)** Microbial alpha diversity in each group was calculated using four different parameters: PD_whole_tree, Chao1, Shannon and Simpson. *p*-value was evaluated with a non-parametric *T*-test. “*” indicates statistically significant (*p*-value < 0.05) differences between groups, and “**” indicates *p*-values < 0.01. PCoA plots of unweighted **(B)** and weighted UniFrac distances **(C)** between groups. **(D)** A histogram of logarithmic (LDA) scores computed for features differentially abundant between groups. LEfSe identifies which microbial clades amongst those detected as statistically differential will explain the greatest differences among the communities, where the black bar represents those of greater abundance in the control group, the blue bar for those in the LD group, and the red bar for those in the HD group. The proportions of *Citrobacter* **(E)** and *Lachnoclostridium* **(F)** in the three groups. The groups were named according to the supplemented *C. freundii* dose: Control = 0, LD = low, and HD = high.

The cecal content samples of nine mice per group (three groups totally) were analyzed. Diversity analysis revealed an intricate relationship between the gut bacterial richness and *C. freundii* concentration in the feed. Overall, mice fed with *C. freundii* showed a significant decrease in bacterial diversity compared to controls (*p-*value < 0.05; non-parametric *t*-test of alpha diversity with parameters including Shannon and Simpson) (Figure 1A).

Multivariate analysis to determine the effect of *C. freundii* supplementation and its concentration on bacterial composition (beta diversity analysis) showed that supplementation of *C. freundii* had a significant impact on the bacterial community structure in mice when it was evaluated with the weighted UniFrac distance (*p-*value = 0.02, PERMANOVA non-parametric test), but not with the unweighted UniFrac distance (*p-*value = 0.23, PERMANOVA non-parametric test) (Figures 1B,C).

To characterize the bacterial homeostasis in mice after *C. freundii* supplementation, we determined the specific taxon that significantly fluctuated in different samples. The standard commensal gut microbiota classes, dominated the gut microbiota of the control group samples: Clostridia (38.9%), Bacteroidia (37.1%), Bacilli (12.2%), and Erysipelotrichia (6.1%). Exposure to a low dose of *C. freundii* induced significant fluctuations of classes Clostridia (decreased to 19.3%) and Bacilli (increased to 28.5%). The exposure to a high dose of *C. freundii* induced significant fluctuations of classes Gammaproteobacteria (increased to 7.2%) and Erysipelotrichia (increased to 32.7%) (Figure 1D). As expected, the genus *Citrobacter* exhibited the highest relative abundance in each sample from the group fed with a high dose of *C. freundii*. The proportion of *Lachnoclostridium* decreased significantly in each sample in the groups fed with *C. freundii* (Figures 1E,F). PICRUSt-prediction revealed significantly more abundant genes associated with lipid metabolism in both experimental groups, and energy metabolism in the HD group, in comparison to the control samples (*p-*value < 0.05) (Supplementary Figure 1).

The clean data obtained by the Illumina and Nanopore sequencing were 1.47 and 6.4 Gb in size, respectively. The complete genome size assembled from these clean data is 4.9 Mb. We identified 4,993 protein coding genes from the genome (Supplementary Table 2). We found two bile acids-related genes in the genome (bile acid: sodium symporter family protein).

### Fluctuation of Bile Acids in the Gut Content of Grass Carp and Mice

**FIGURE 2:**
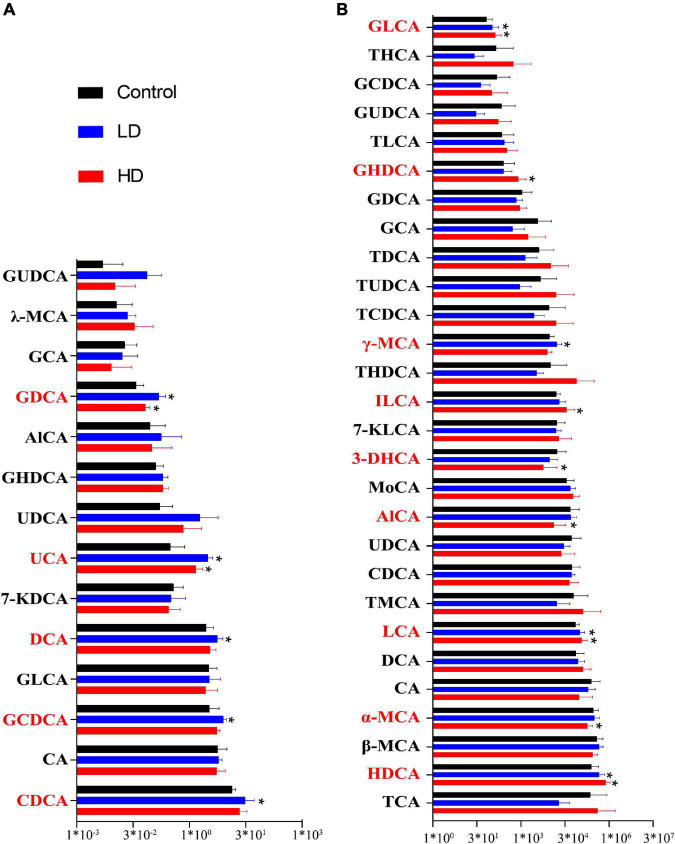
Fluctuation of the gut content bile acids in grass carp **(A)** and mice **(B)**. The “*” indicates statistically significant (*p*-value < 0.05) differences compared with the control group.

Bile acid (BA) content was determined in the gut samples of both grass carp (6 samples per group) and mice (10 gut samples per group) using HPLC-MS. In grass carp, we found 14 BA species, with Chenodeoxycholic acid (CDCA), Cholic acid (CA) and Glycochenodeoxycholic acid (GCDCA) being the most dominant. Five BA species significantly differed (*p* < 0.05, pairwise *T*-test) between the experimental and control groups: Ursocholic acid (UCA), Glycodeoxycholic acid (GDCA), DCA, GCDCA and CDCA (Figure 2A).

In mice, we found 28 BA species. The most abundant BA was Hyodeoxycholic acid (HDCA), followed by the CA, Taurocholic acid (TCA), α-Muricholic acid (α-MCA), and β-Muricholic acid (β-MCA). Primary bile acid α-MCA was lower in the experimental groups. Several secondary bile acids, including Glycolithocholic acid (GLCA), LCA, HDCA and Isolithocholic acid (ILCA), were significantly higher in the experimental groups, whereas 3-Dehydrocholic acid (3-DHCA) was significantly lower in the experimental groups (*p* < 0.05, pairwise *T*-test) (Figure 2B).

### *Citrobacter freundii* GC01 Induced Multimodal Responses in Lipid Metabolism-Associated Genes in Grass Carp

We sequenced transcriptomes from liver tissues of 5 grass carp specimens from each diet group to infer their gene expression profiles. RNA sequencing produced about 111.1 million (Control), 104.5 million (LD), and 105.3 million (HD) raw reads for the three groups. After filtration, 109.1 million (Control), 102.4 million (LD) and 103.0 million (HD) raw reads were obtained and these sequences were carried forward for additional analysis. On average, 85% of the clean reads were mapped to the reference genome.

When compared with the control group, 3,257 transcripts were labeled as significantly differentially expressed genes (DEGs). Among these, 2,579 were in the LD group and 1,187 were in the HD group. The 2,579 DEGs in the LD group included 686 up-regulated transcripts [fold change (fc) ≥ 2, *p* < 0.05] and 342 down-regulated transcripts (fc ≤ 0.5, *p* < 0.05). The 1,187 DEGs in the HD group included 356 up-regulated and 176 down-regulated.

**FIGURE 3:**
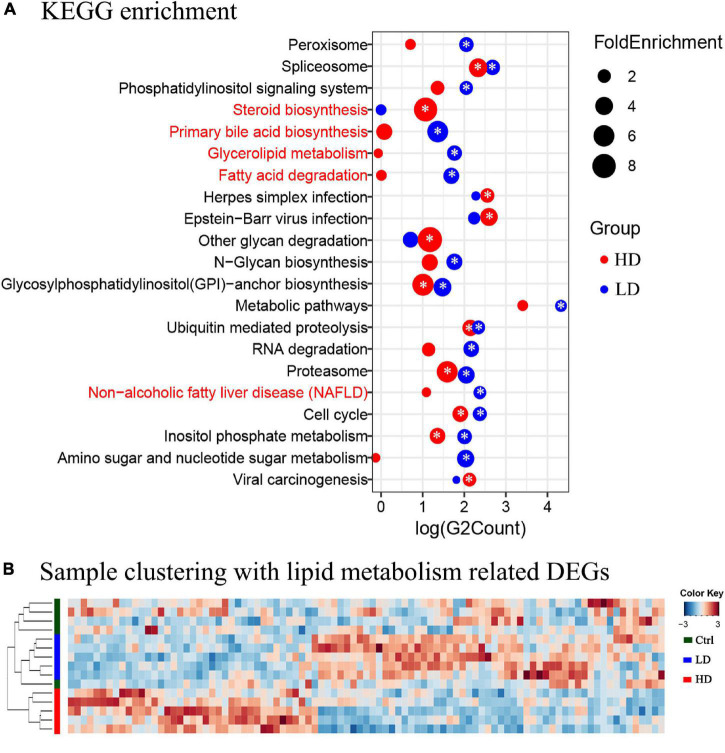
**(A)** The KEGG enrichment analysis of the grass carp liver transcriptome data. The *y*-axis shows the names of pathways. The names of metabolism-related pathways are marked by the red font. The *x*-axis shows the log value of DEGs count in each pathway. The blue circles indicate enriched DEGs between the LD and control groups, and the red circles indicate enriched DEGs between the HD and control groups. The size of each circle corresponds to the magnitude of fold-change. The “*” symbol in the middle of circles indicates that enrichment in that pathway was significant when compared with the control group (*p* value < 0.05). **(B)** Heatmap and clustering of DEGs associated with lipid metabolism in liver samples. Cluster analysis performed on Bray-Curtis distance matrices of DEGs using the unweighted pair group mean algorithm (UPGMA). The colored bars to the left indicate the group identity of samples: the green bar represents the control group, the blue bar represents the LD group, and the red bar represents the HD group.

The KEGG pathway enrichment analysis was applied to help us further understand the biological functions of DEGs in the liver samples; 2,579 DEGs in the LD group were significantly enriched in 16 KEGG pathways and 1,187 DEGs in the HD group were significantly enriched in 10 KEGG pathways (Figure 3A). Three lipid metabolism-related pathways, primary bile acid biosynthesis, glycerolipid metabolism, fatty acid degradation, and non-alcoholic fatty liver disease (NAFLD), were enriched in both experimental groups, but significantly only in the LD group.

In order to further understand the expression profiles of the lipid metabolism-related genes in the livers of three groups, the FPKM values of all 93 DEGs related to lipid metabolism were analyzed using Heatmap. The samples formed two clusters: one contained all 5 samples from the Control group, and the other contained all samples from the LD and HD groups (Figure 3B).

### Validation of Candidate Genes

**FIGURE 4:**
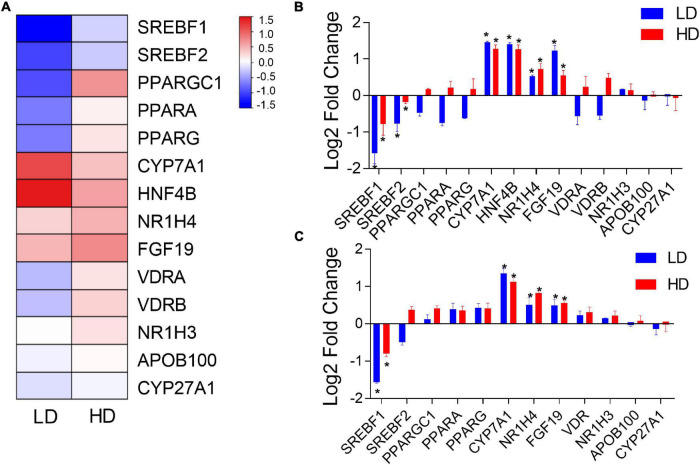
**(A)** Hierarchical clustering heatmap of transcriptional analysis of lipid metabolism-related gene expression in the grass carp liver. The two experimental groups are on the *x*-axis, whereas names of lipid metabolism-related genes are on the *y*-axis. The expression of lipid metabolism-related genes in the livers of grass carp **(B)** and mice **(C)** inferred using qPCR. The *x*-axis shows the names of genes, and the *y*-axis indicates the Log 2 fold change with the relative expression (compared to the Control group) of each gene. * indicates statistically significant (*p*-value < 0.05).

To compare the impacts of *C. freundii* supplementation on the transcription of genes between mice and grass carp (and corroborate the transcriptomic results in grass carp), 14 lipid metabolism-related grass carp DEGs were selected for validation by qPCR: *CPY7A1*, *CPY27A1*, *NR1H4*, *NR1H3*, *HNF4B*, *PPARA*, *PPARG*, *PPARGC1*, *VDRA*, *VDRB*, *SREBF1*, *SREBF2*, *APOB100*, and *FGF19*. In mouse samples, we could test only 12 of these genes, because *HNF4B* has not been identified in the mouse genome, whereas grass carp paralogs *VDRA* and *VDRB* have only one ortholog in the mouse genome: *VDR*. Figure 4A presents a hierarchical clustering heatmap of transcriptional analysis of expression of lipid metabolism-related genes in the grass carp liver. The qPCR results in grass carp samples were fully congruent with transcriptomics results, thereby indirectly confirming the validity of the RNA-seq analysis. Results were only partially congruent between the grass carp and mouse samples: *CYP7A1*, *NR1H4* and *FGF19* were significantly up-regulated, and *SREBF1* was significantly down-regulated in the livers of both grass carp and mice fed a diet supplemented with *C. freundii* (Figures 4B,C). In grass carp samples, the *HNF4B* gene was also significantly up-regulated, while *SREBF2* was significantly down-regulated (Figure 4B).

### Testing the Association Between *HNF4B* and *CYP7A1* in Grass Carp

As transcriptomics analysis indicated that *HNF4B* and *CYP7A1* genes were both significantly up-regulated in grass carp, we further verified this association via an overexpression test. We transfected the constructed full-length recombinant *HNF4B* plasmids from grass carp into CIK cells for transient overexpression and analyzed the expression of *CYP7A1*. Results showed that the expression of *CYP7A1* in cells that exhibited transient overexpression of *HNF4B* was significantly increased at 1dpf and 3dpf (Supplementary Figure 2).

### Oil Red-O Staining of Grass Carp Liver Sections

**FIGURE 5:**
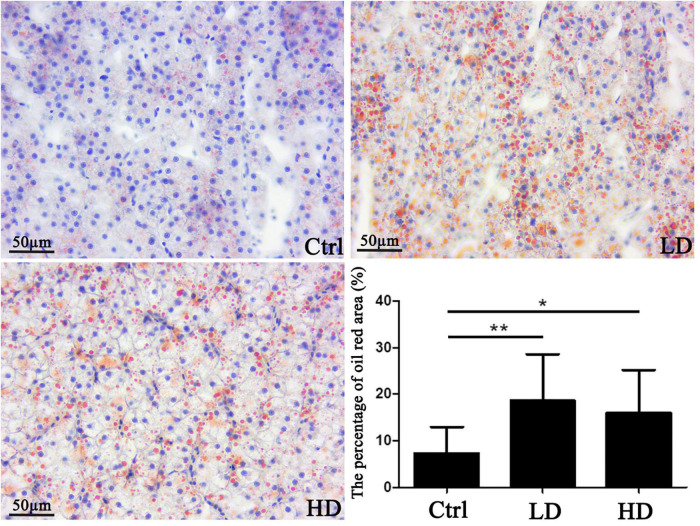
Hepatic lipid accumulation in a grass carp fed a diet supplemented with *Citrobacter freundii* GC01: visualization and quantification of lipids in grass carp liver samples using the Oil red-O staining. Scale bar = 50 μm. The small blue spots are nuclei (blue arrows), whereas the red nearly spherical dots are lipid droplets (black arrows). * *p*-value < 0.05 compared with the control group. Values represent means ± sem. **p-value < 0.01 compared with the control group.

The analysis of oil red-O stained liver sections revealed that the areas of lipid droplets were 7.3 ± 6.5% in the control, 18.6 ± 13.1% in the LD, and 15.9 ± 17.8% in the HD group. Statistical analysis showed that areas of lipid droplets of experimental groups (LD and HD group) were significantly higher than that of the control group (*p* value < 0.05) (Figure 5).

### Serum Biochemical Values in Grass Carp

**FIGURE 6:**
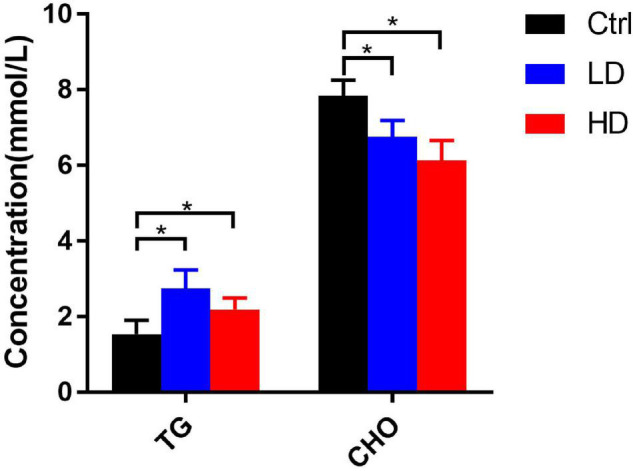
Changes of serum biochemical indexes in different experimental groups of grass carp. * indicates statistically significant (*p*-value < 0.05).

Generally, the serum triglycerides contents in the LD and HD groups were significantly higher than in the control group (*p*-value < 0.05) (Figure 6). The total cholesterol contents in the LD and HD groups were significantly lower than in the control group (*p-*value < 0.05) (Figure 6).

## Discussion

The intestinal microbiota has been widely recognized as an integral part of the host that plays an important role in host physiology, including the energy metabolism (Rawls et al., 2004; Flint et al., 2008). The imbalance of energy metabolism may induce obesity, which in turn often causes a variety of diseases, so it has become a serious health problem in contemporary human societies. Recent research suggested that a diet-caused intestinal microbiota alteration may induce obesity (Turnbaugh et al., 2008; Zhang et al., 2020). However, the mechanism by which microbiota cause obesity is unclear, despite the increasing scientific attention that it attracted in recent years. Here, we fed grass carp and mice with *C. freundii* GC01 and investigated the fluctuation of intestinal microbiota and bile acid-associated metabolites. We found that regulation of bile acid metabolism by the intestinal microbiome is evolutionarily highly functionally conserved between higher and lower vertebrates.

We found that CDCA, CA, and GCDCA were dominant bile acids in the grass carp intestine, which is common in fish (Zhang et al., 2017; Romano et al., 2020). When *C. freundii* was supplemented to the grass carp feed, the abundance of two primary bile acids (GCDCA and CDCA) and three secondary bile acids (GDCA, UCA, and DCA) was significantly affected. It is unclear whether this effect was achieved directly by changing the abundance of *C. freundii*, or indirectly via its impact on the overall composition of gut microbiota. The identification of two bile acids-related genes in the genome of this bacterial strain indicates that it may be able to affect the bile acids composition directly, but it is possible that both variables played a role. The significantly higher abundance of primary bile acids in experimental groups is consistent with the lower total cholesterol content in these two groups. Our results revealed that TCA, HDCA, α-MCA, β-MCA, and CA were dominant bile acids in mice, which is generally also consistent with some previous results (Vallim et al., 2013; Zheng et al., 2017). The dietary supplementation of *C. freundii* significantly affected one primary (α-MCA) and five secondary (GLCA, LCA, HDCA, ILCA, and 3-DHCA) bile acids in mice. The significant changes of primary bile acids and secondary bile salts both in grass carp and mice fed on a diet supplemented with *C. freundii* suggest that it influences the bile acid metabolism. This is indirectly further supported by the finding of two bile acids-related genes in the genome of *C. freundii.*

Primary bile acids are synthesized in the liver from cholesterol by up to 17 enzymes, with cholesterol 7a-hydroxylase (CYP7A1) being the first rate-limiting enzyme of the “classic biosynthesis pathway” in mice (Vallim et al., 2013; Hofmann and Hagey, 2014). Despite the *CYP7A1* up-regulation, we did not find a significant increase in the cecal content of primary bile acids in mice. However, we found that primary bile acid α-MCA was significantly lower, and secondary bile acid HDCA was significantly higher in mice fed with *C. freundii*. α-MCA can be transformed into HDCA by intestinal microbiota in rats (Eyssen et al., 1999). Here, the significantly higher HDCA and lower α-MCA in experimental groups might be indicative of biotransformation by *C. freundii*. In addition, the analysis of the cecal content conducted herein indicates that almost all primary bile acids are reabsorbed before the intestinal content reaches the cecum, and recirculated back into the liver through the enterohepatic circulation (Ridlon et al., 2006). This may be the mechanism that explains the absence of an increase in primary bile acids.

The long-known important role of *CYP7A1* in hepatic bile salt synthesis (Inoue et al., 2006) has been confirmed in zebrafish as well, where it was identified as the gene that encodes the rate-limiting enzyme, cholesterol 7alpha-hydroxylase, and possibly induces the synthesis of primary bile acids (Wen et al., 2021). *HNF4* is a family of transcription factors that are crucial for maintaining the bile acid, lipid, and glucose homeostasis in the liver, also known to be associated with the *FXR* in mammals and zebrafish (Inoue et al., 2006; Wen et al., 2021). In the present study, we found that *CYP7A1* was significantly up-regulated in grass carp and that the overexpression of *HNF4B* from grass carp resulted in a significant increase in the expression level of *CYP7A1*. We also found that primary bile acids GCDCA and CDCA were significantly more abundant in grass carp fed on *C. freundii* compared with the control group. All these results suggest that *C. freundii* may regulate the bile salt synthesis through *CYP7A1* in grass carp. Generally, our results agree with the proposals of Sayin et al. (2013) and Wen et al. (2021) that the gut microbiota not only regulates the secondary bile acid metabolism but also regulates the bile acid synthesis in the liver.

In the present study, analysis of liver transcriptome sequencing data disclosed that the lipid metabolism-related pathways, primary bile acid biosynthesis, fatty acid degradation and NAFLD, were enriched in both experimental groups. PICRUSt-prediction revealed significantly more abundant genes associated with lipid metabolism in bacterial groups. Oil red-O staining of liver sections revealed that areas of lipid droplets of experimental groups were significantly higher than that of the control group, indicating increased fat accumulation. In addition, the serum triglycerides contents in experimental groups were significantly higher than in the control group. The bile acid profile was different between the experimental and control groups, especially several secondary bile acids were significantly. All these results suggest that *C. freundii* GC01 probably influenced lipid metabolism in a bile acid-dependent manner. Our results are generally consistent with those of Zheng et al. (2017), who found that bile acids were the most significant factor correlated with the intestinal microbiota, and that supplementing bile acids to mice fed a standard diet induced an obese phenotype, biochemical parameters similar to those observed in a high-fat diet-fed mice, as well as increased liver weight and total serum cholesterol. Similarly, Parséus et al. (2017) found that microbiota in mice intestine promoted diet-induced obesity and associated phenotypes through FXR, and the changes in the bile acid profile might partly explain the increased adiposity.

When *C. freundii* GC01 was supplemented to the grass carp diet, *CYP7A1* and *HNF4B* genes were significantly up-regulated. The overexpression of *HNF4B* also resulted in a significant increase in the expression of *CYP7A1*. This gene plays a central role in regulating the bile acid biosynthesis in mice and zebrafish (Vallim et al., 2013; Wen et al., 2021). Thus, *HNF4B* might be an important regulator in BA metabolism in grass carp.

## Conclusion

Together, these results suggest that despite the wide evolutionary gap between the grass carp and mouse, *C. freundii* GC01 regulates the bile acid metabolism in both species, i.e., the role of intestinal microbiota in regulating the metabolism of bile acids is conserved across vertebrates. Elucidating the roles of intestinal microbiota in the bile acid metabolism will expand our understanding of the physiological functions of intestinal microbiota and provide new insights into the mechanisms underlying the intestinal commensal-host relationships.

## Data Availability Statement

The datasets presented in this study can be found in online repositories. The names of the repository/repositories and accession number(s) can be found below: https://www.ncbi.nlm.nih.gov/, PRJNA580128; https://www.ncbi.nlm.nih.gov/, PRJNA740072; and https://www.ncbi.nlm.nih.gov/, PRJNA792138.

## Ethics Statement

The animal study was reviewed and approved by Institute of Hydrobiology, Chinese Academy of Sciences.

## Author Contributions

SW, GW, and FX conceived and designed the experiments. FX performed the experiments. FX, SC, SW, and IJ wrote the manuscript. WL, ML and HZ edited the manuscript. All authors read and approved the final manuscript.

## Conflict of Interest

The authors declare that the research was conducted in the absence of any commercial or financial relationships that could be construed as a potential conflict of interest.

## Publisher’s Note

All claims expressed in this article are solely those of the authors and do not necessarily represent those of their affiliated organizations, or those of the publisher, the editors and the reviewers. Any product that may be evaluated in this article, or claim that may be made by its manufacturer, is not guaranteed or endorsed by the publisher.

## Footnotes

1
http://qiime.org/

2
http://huttenhower.sph.harvard.edu/galaxy/

## Abbreviations

*C. freundii*: *Citrobacter freundii*
FXR: farnesoid X receptor
BA(s): bile acid(s)
Ctrl group: control group
LD group: a diet supplemented with 0.5*10^7^cfu/g *C. freundii* GC01
HD group: a diet supplemented with 10^7^cfu/g *C. freundii* GC01
OTU: Operational taxonomic unit
PCoA: Principal coordinate analysis
LEfSe: Linear discriminant analysis Effect Size
FPKM: fragments per kilobase of exon per million fragments mapped
DEGs: differentially expressed genes
qPCR: real-time quantitative PCR
ORO: Oil red-O
HPLC-MS: high-performance liquid chromatography-mass spectrometry
NAFLD: non-alcoholic fatty liver disease
CDCA: Chenodeoxycholic acid
CA: Cholic acid
GCDCA: Glycochenodeoxycholic acid
UCA: Ursocholic acid
GDCA: Glycodeoxycholic acid
DCA: deoxycholic acid
HDCA: Hyodeoxycholic acid
TCA: Taurocholic acid
α-MCA: α-Muricholic acid
β-MCA: β-Muricholic acid
GLCA: Glycolithocholic acid
LCA: lithocholic acid
ILCA: Isolithocholic acid
GLCA: Glycolithocholic acid
LCA: lithocholic acid
ILCA: Isolithocholic acid
3-DHCA: 3-Dehydrocholic acid.
