# Supplementary material for: The Role of Intestinal Microbiota in Regulating the Metabolism of Bile Acids Is Conserved Across Vertebrates
Source: Front Microbiol. 2022 Feb 15;13:824611. doi: 10.3389/fmicb.2022.824611 (PMC8887563; doi:10.3389/fmicb.2022.824611)
Supplement: Supplementary file 2 [file Data_Sheet_2.docx]

**Supplementary Files**

Table S1.

Primers used for qPCR analysis in grass carp taken over from previous studies, and associated references (ref).

| Gene name | Forward primer | Reverse primer | ref |
| --- | --- | --- | --- |
| *SREBF1* | GAGTCTTGGGGTTGGATGGA | TGAGACACGTCCAGAGGTTC | ref 1 |
| *CYP7A1* | TCTATGACAATCCTCTGGCATACAA | CAAAGAAACGGCCCGGACA | ref 2 |
| *NR1H4* | CGTCCAGGTGTTGGTTGAGT | CGAACGTAGGAACATCGCCT | ref 2 |
| *CYP27A1* | ACCAGCGGTCTGGAACAAAA | AAGAGCAGCCGAACACAGAA | ref 2 |
| *β-actin* | ACCCTGAAGTACCCCATCGA | CAGAGGCATACAGGGACAGC | ref 1 |
| *gapdh* | GGGAAACTGTGGAGGGATGG | TGCAGCCTTGACCACTTTCT | ref 1 |

Primers used for qPCR of grass carp genes newly designed for this study.

| Gene name | Forward primer | Reverse primer | CDS in grass carp genome |
| --- | --- | --- | --- |
| *SREBF2* | ATCTCAGCTGTCTGCCTGGT | GGCGGAGTCTTCAGTACAGC | CI01000214_00365143_00371990 |
| *PPARGC1* | GAGAGACAACGGCAAAAAGC | ATCTCGCCGAAGACTTCAAA | CI01000043_01383746_01406582 |
| *PPARA* | GAAAGCATGGCTTCTTCAGG | CTCTGCTTTCAGCCTCTGCT | CI01000020_00798124_00809552 |
| *PPARG* | GATACAGAGCGCACCAGACA | CGAACCGTTTCTGTGAGTGA | CI01000057_05596837_05615278 |
| *HNF4B* | TGGAAGAGCTGGTGAGACCT | ACAACAACAGCTCCCCAAAC | CI01000043_02555479_02574821 |
| *FGF19* | ATGCAAAGCTCGGACAGTTT | CAGTCCCGTCTTTACCCAAA | CI01000043_04465956_04468671 |
| *VDRA* | CGTCAATGATGTCACCAAGG | GCAGCACATGTTCCTCTTCA | CI01000129_01449221_01458138 |
| *VDRB* | ACTCCTTCAGCCACTCTCCA | CACTGGACTTCAGCAAAGCA | CI01000340_08808101_08822062 |
| *NR1H3* | AAGGCATGAATGACCTCCAC | AGCATACGGGGAAACATCAG | CI01000026_07853925_07863410 |
| *APOB100* | TCAAGAGTTGAGCGGAAGGT | GTACAACTCCTGCCCGGTAA | CI01000189_02989005_03008501 |

Primers used for qPCR analysis in mice taken over from previous studies, and associated references (ref).

| Gene name | Forward primer | Reverse primer | ref |
| --- | --- | --- | --- |
| *SREBF1* | TCTGCCTTGATGAAGTGTGG | AGCAGCCCCTAGAACAAACA | ref 3 |
| *PPARA* | TCGAGGAAGGCACTACACCT | TCTTCCCAAAGCTCCTTCAA | ref 3 |
| *PPARG* | ACGATCTGCCTGAGGTCTGT | CATCGAGGACATCCAAGACA | ref 3 |
| *CYP7A1* | CAAGAACCTGTACATGAGGGAC | CACTTCTTCAGAGGCTGCTTTC | ref 4 |
| *NR1H4* | ACAGCTAATGAGGACGACAG | GATTTCCTGAGGCATTCTCTG | ref 5 |
| *GAPDH* | AAATGGTGAAGGTCGGTGTG | CATGTAGTTGAGGTCAATGAAGG | ref 6 |
| *36B4* | GCCCTGCACTCTCGCTTTCT | CAACTGGGCACCGAGGCAACAGTTG | ref 4 |

Primers used for qPCR of grass carp genes newly designed for this study.

| Gene name | Forward primer | Reverse primer | Accession No |
| --- | --- | --- | --- |
| *FGF19* | CAGTCTGTGCCAGGTGAAGA | CTGAAGGTGCAGTCCTCCTC | XM_021216925.2 |
| *VDR* | GAGGTGTCTGAAGCCTGGAG | ACCTGCTTTCCTGGGTAGGT | NM_009504.4 |
| *NR1H3* | CTGCAGGACAAAAAGCTTCC | CCCTTCTCAGTCTGCTCCAC | NM_001355279.1 |
| *APOB100* | AGCTTGCTCAAGGAGAGCTG | TCAGGCTGCTTTGAAGGTCT | NM_009693.2 |
| *CYP27A1* | TGAAACCCTCCATTCCTGAG | AGGAAGTGCAGGTAGCCAGA | NM_024264.5 |
| *SREBF2* | CCATCTTCCCCTCTCTTTCC | AGGGAAGATCCTGGGAGAAA | NM_033218.1 |
| *PPARGC1* | CCGAGAATTCATGGAGCAAT | TTTCTGTGGGTTTGGTGTGA | NM_008904.2 |

Body weight gain data were analyzed statistically with One-way ANOVA implemented in SPSS 16.0. We did not find any significant difference (F=1.476, *P*=0.240).

**References**

Ref 1: Xu, Y. H., Tan, X. Y., Xu, Y. C., Zhao, T., Zhang, L. H., and Luo, Z. (2019). Novel insights for SREBP-1 as a key transcription factor in regulating lipogenesis in a freshwater teleost, grass carp *Ctenopharyngodon idella*. *Br. J. Nutr.* 122, 1201-1211. doi: 10.1017/S0007114519001934

Ref 2: Tian, J. J., Jin, Y. Q., Yu, E. M., Sun, J. H., Xia, Y., Zhang, K., et al. (2021). Farnesoid X receptor is an effective target for modulating lipid accumulation in grass carp, *Ctenopharyngodon idella*. *Aquaculture* 534:736248. doi: 10.1016/j.aquaculture.2020.736248

Ref 3: Magliano, D. A. C., Bargut, T. C. L., de Carvalho, S. N., Aguila, M. B., Mandarim-de-Lacerda, C. A., and Souza-Mello,V. (2013). Peroxisome proliferator-activated receptors-alpha and gamma are targets to treat offspring from maternal diet-induced obesity in mice. *PLoS ONE* 8:e64258. doi: 10.1371/journal.pone.0064258

Ref 4: Zhang, L. S., Huang, X. F., Meng, Z. P., Dong, B. N., Shiah, S., Moore, D. D., et al. (2009). Significance and mechanism of CYP7a1 gene regulation during the acute phase of liver regeneration. *Mol. Endocrinol.* 23, 137-145. doi: 10.1210/me.2008-0198

Ref 5: Massafra, V., Milona, A., Vos, H. R., Ramos, R. J. J., Gerrits, J., Willemsen, E. C. L., et al. (2017). Farnesoid X receptor activation promotes hepatic amino acid catabolism and ammonium clearance in mice. *Gastroenterology* 152, 1462-1476. doi: 10.1053/j.gastro.2017.01.014

Ref 6: De Souza, A. T., Dai, X. D., Spencer, A. G., Reppen, T., Menzie, A., Roesch, P. L., et al. (2006). Transcriptional and phenotypic comparisons of *Ppara* knockout and siRNA knockdown mice. *Nucleic Acids Res.* 34, 4486-4494. doi: 10.1093/nar/gkl609


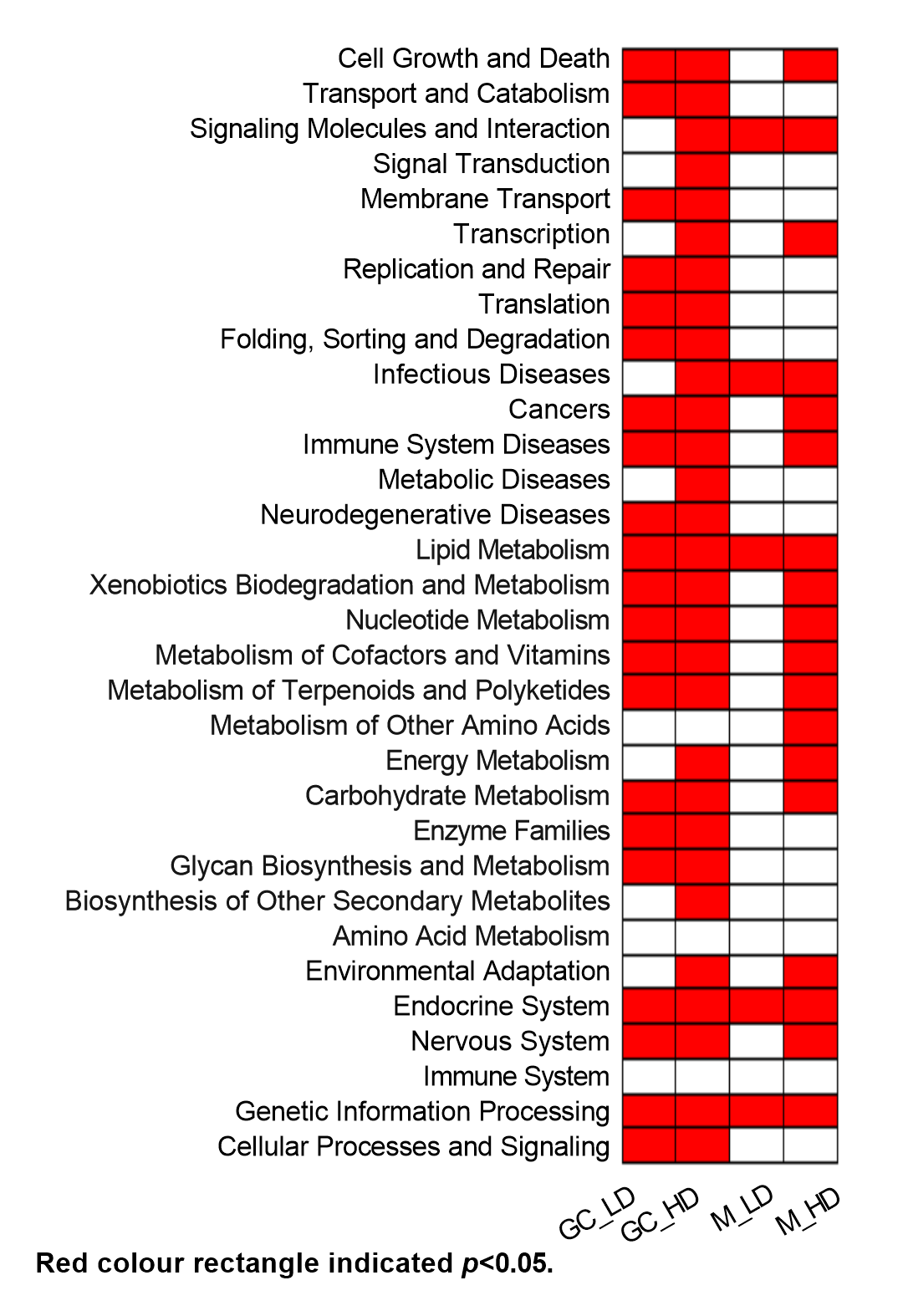


**Figure S1.** PICRUSt functional prediction of the intestinal microbiota in grass carp and mouse. The red colour indicates statistically significant (*p*-value<0.05) differences compared with thecontrol group. GC stands for grass carp, M stands for mice, whereas LD and HD are diet groups.


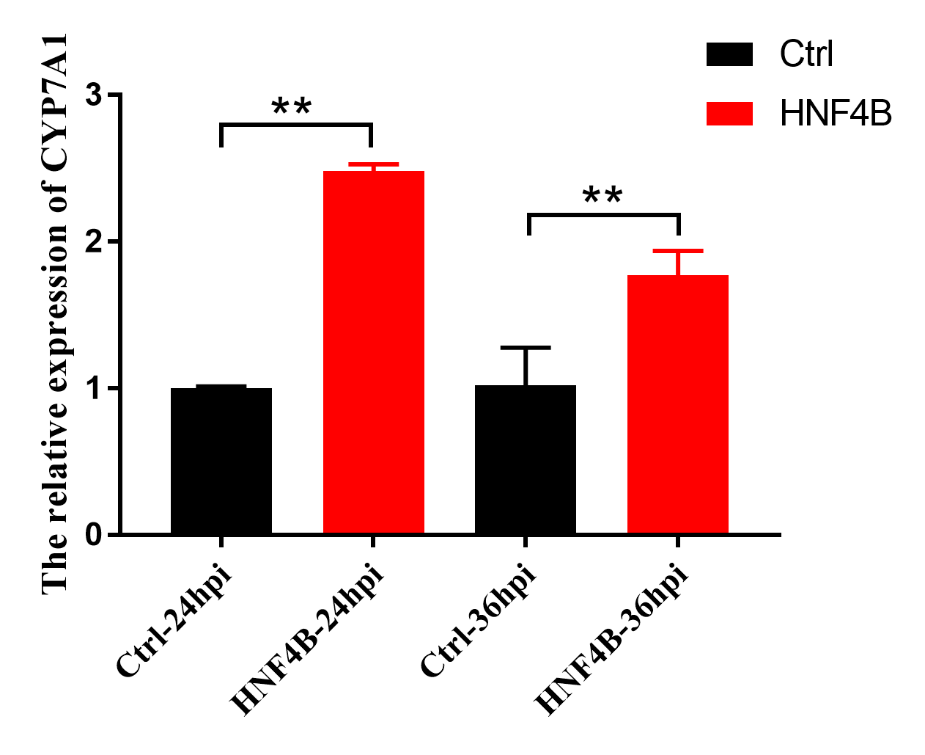


**Figure S2.** The effect of overexpression of *HNF4B* on *CYP7A1* expression.
